# 12th Annual Biotechnology Congress (BAC Girona 2018): abstract collection: Girona, Spain. 11-13 July 2018

**DOI:** 10.1186/s12896-018-0469-3

**Published:** 2018-10-29

## Introduction

### Aitor Balmaseda, Juan Calvet

#### Spanish Federation of Biotechnologist, León, 24007, Spain

This year the Spanish Federation of Biotechnologists (https://febiotec.es/) held the 12^th^ edition of the Annual Biotechnology Congress (BAC) in Girona from 11^th^ to 13^th^ of July 2018. What is more, this entity with more than 1700 members, celebrated the 10^th^ anniversary of its foundation.

One of the main goals of the event was to develop the appropriate atmosphere to allow the meeting and results sharing of young students and professionals with the audience and senior professionals. BAC Girona 2018 gathered biotechnologists from all over Spain to learn and enjoy the last trends in the field. This year’s conferences included speeches from academia researchers, business biotechnology and oral communications of our assistants. As a recognition to the work of our participants, we are proud of sharing a sample of the abstracts presented in Girona.

We hope to see you in the following edition of our Congress which will take place in Madrid in July 2019.

## O1 Virtual biopsy: development of non-invasive immunotargeted imaging agents for the diagnosis of glioblastoma

### Eduardo Ruiz-López^1^, Ruth González-Gómez^1^, Beatriz Torres-Herrero^1^, Sara Naya-Forcano^1^, Natalia Magro^2^, Eduardo Romero^2^, Héctor Tejero^3^, Fátima Al-Shahrour^3^, Miguel A. Morcillo^2^, Alberto J Schuhmacher^1^

#### ^1^Molecular Oncology Group, Aragon Health Research Institute (IIS ARAGON), Zaragoza, 50009, Spain; ^2^Biomedical Applications of Radioisotopes and Pharmacokinetics Unit, Research Centre for Energy, Environment and Technology (CIEMAT), Madrid, 28040, Spain; ^3^Bioinformatics Unit, Spanish National Cancer Research Center (CNIO), Madrid, 28029, Spain

##### **Correspondence:** Eduardo Ruiz-López (eruiz@iisaragon.es)

Glioblastoma (GBM) is the most common and aggressive brain tumor. Current diagnosis of GBM by Magnetic Resonance Imaging (MRI) provides morphological, sometimes inaccurate, information. A brain biopsy is finally often required [1]. One alternative is Positron Emission Tomography (PET) but, unfortunately, the most widely used tracer, ^18^F-Fluorodeoxyglucose (^18^F-FDG), is ineffective due to the high consumption of glucose by the brain [2].

An innovative option is termed “immunotargeted imaging” [3]. By merging the high target selectivity and specificity of antibodies with the high spatial resolution, sensitivity, and quantitative capabilities of PET, it is possible to conduct the non-invasive diagnosis and monitoring of patients over time using *in vivo*, integrated, quantifiable, 3D, full body “immunohistochemistry”. ImmunoPET could be considered a virtual biopsy.

Applying bioinformatics on patient datasets, we have identified molecular targets for the development of an immunoPET agent for GBM, due to the high levels of protein expression in GBM and negligible levels in normal brain. We are developing novel immunoPET agents against these candidates using murine monoclonal antibodies labeled with ^89^Zirconium in xenograft mice and “avatar” models. For clinical development, we are reducing the radioactivity exposure and optimizing the blood brain barrier permeability of the tracers by generating different antibody fragments with faster clearance and pharmacokinetics [4]. As matching of the physical half-life of the positron-emitting radionuclide with the biological half-life of the antibody or fragment is crucial for immunoPET, these smaller derivatives will be labeled with PET isotopes of shorter half-lives, such as ^68^Galium, which advantageously can be produced in a generator rather than a cyclotron. Click chemistry by biorthogonal reactions *in vivo* allows the matching of other antibodies/fragments with tracers and thereby diminishes the exposure to radioactivity to ensure a better signal-to-noise ratio. We are exploiting this approach to label multiple imaging tracers, including MRI-tracers, to the same pre-targeted molecule *in vitro* as well as multi-modal and multifunctional imaging and theragnostics. These imaging agents could be used for other tumor types and pathologies and may have a major impact on the diagnosis and monitoring of patients.

References

1. Ahmed R, *et al.* Malignant gliomas: current perspectives in diagnosis, treatment, and early response assessment using advanced quantitative imaging methods. *Cancer management and research.* 2014; 6:149-70.

2. La Fougère C, *et al.* Molecular imaging of gliomas with PET: opportunities and limitations. *Neuro Oncology*. 2011; 13:806-19.

3. De Lucas A G, Schuhmacher A J, *et al.* Targeting MT1-MMP as an ImmunoPET-Based Strategy for Imaging Gliomas. PLoS One. 2016; 11(7):e0158634.

4. Freise A C, Wu A M. In vivo imaging with antibodies and engineered fragments. *Molecular Immunology*. 2015; 67(2 Pt A):142-52.

Funding:

Asociación Española Contra el Cáncer (AECC). Programa Ramón y Cajal (RYC). Fundación de Investigación Oncológica (FERO)

## O2 Characterization of photoswitchable hydrophobic and amphiphilic peptides for folding and membrane insertion studies

### Mónica V. Gutiérrez-Salazar^1^, Diego Sampedro^2^, Eduardo Santamaría^2^, Victor Lórenz-Fonfría^1^

#### ^1^ Membrane Biophysics, Instituto de Ciencia Molecular (ICMol), Universitat de València, Paterna, 46980, Spain; ^2^ Department of Chemistry, Centro de Investigación en Síntesis Química (CISQ), Universidad de la Rioja, Logroño, 26006, Spain

##### **Correspondence:** Mónica V. Gutiérrez-Salazar (movicgu@uv.es)

Mechanics and kinetics of folding and insertion of hydrophobic and amphiphilic peptides in lipidic membranes is a process not fully understood. This is mainly due to the lack of sensitive experimental procedures that allow to follow the process with sufficient temporal and structural resolution. A practical approach to achieve this sensitivity is photocontrol of folding, using molecular photoswitches linked to the peptide. These photoswitches isomerize when irradiated with light of specific wavelengths, and can induce changes in the structure of the peptide, as folding or unfolding of the peptide and, consequently, modulate the insertion in the membrane. This work aims to characterize two model peptides (hydrophobic and amphiphilic) linked to a derivate of the molecular photoswitch azobenzene, by circular dichroism, infrared and ultraviolet/visible spectroscopies, to use them in future folding and membrane insertion studies. We designed different lighting setups to achieve photoisomerization with precision and in controlled environment. We characterized the azobenzene derivative (BCA) and concluded that it is a suitable candidate for photoswitching. The photoswitchable peptides were characterized in membrane-mimicking environments and solvents, to know in which conditions their structure could be controlled with photoswitching. The hydrophobic peptide showed good photoisomerization of BCA, but poor control of structure in most solvents. We managed to photocontrol the structure of the amphiphilic peptide in solvents, and some promising results were obtained in membrane environments. This work aims to be taken as a starting point for finding suitable photoswitchable membrane peptides.

References

1. Gelman H, Gruebele M. Fast protein folding kinetics. Q. Rev. Biophys. 2014; 47, 95–142

2. Ulmschneider JP, Andersson M, Ulmschneider MB. Determining peptide partitioning properties via computer simulation. J. Membr. Biol. 2011; 239, 15–26

3. Szymański W, Beierle JM, Kistemaker HAV, Velema WA, Feringa BL. Reversible photocontrol of biological systems by the incorporation of molecular photoswitches. Chem. Rev. 2013; 113, 6114–6178

4. Flint DG, Kumita JR, Smart OS, Woolley GA. Using an azobenzene cross-linker to either increase or decrease peptide helix content upon Trans-to-cis photoisomerization. Chem. Biol. 2002; 9, 391–397

Funding:

BFU2016-76805-P, Ministerio de Economía, Industria y Competitividad

## O3 Inhibition of HIF-1α/BNIP3 axis and hypoxia-mediated mitophagy by melatonin addition enhances human hepatocellular carcinoma cells sensitivity to sorafenib treatment

### Flavia Fondevila^1,2^, Carolina Méndez-Blanco^1,2^, Néstor Prieto-Domínguez^1,2^, Paula Fernández-Palanca^1,2^, Andrés García-Palomo^3^, Javier González-Gallego^1,2^, José Luis Mauriz^1,2^

#### ^1^ Institute of Biomedicine (IBIOMED), University of León, León, Spain; ^2^ Centro de investigación biomédica en red de enfermedades hepáticas y digestivas (CIBERehd), ISCIII, Madrid, Spain; ^3^ Medical Oncology Service, Complejo Asistencial Universitario de León, Hospital of León, León, Spain

##### **Correspondence:** Flavia Fondevila (ffonp@unileon.es)

Sorafenib is the only drug approved as first-line treatment for advanced hepatocellular carcinoma (HCC), but its efficacy is limited due to the development of resistant tumor cells. Overexpression of hypoxia-inducible factor 1 alpha (HIF-1α) is related to sorafenib acquired resistance in advanced HCC. The HIF-1α target B-cell lymphoma-2/adenovirus E1B 19 kDa-interacting protein 3 (BNIP3) is the master regulator of hypoxia-induced mitophagy in liver, a process which exhibits a dual role in cancer favoring cell survival or death depending on the cellular context. Melatonin has shown oncostatic, proapoptotic, antiangiogenic and antimetastatic properties on HCC cells.

Our aim was to evaluate the melatonin ability to enhance the sensitivity of HCC cells to sorafenib, focusing on the modulation of HIF-1α-induced mitophagy.

Hep3B human HCC cells were treated with the hypoximimetic CoCl_2_ (100 μM), sorafenib (5 μM) and melatonin (2 mM). siRNAs were employed to silence HIF-1α and BNIP3 genes; cell viability was analyzed by MTT assay; hypoxia, mitophagy and apoptosis-related proteins levels were measured by Western blot; mitochondria and lysosomes colocalization was assayed using immunofluorescence, laser confocal imaging and ImageJ software; and GraphPad Prism 6 software was employed to perform the statistical analysis, considering significant differences when p<0.05.

Coadministration of melatonin and sorafenib strongly reduced the hypoxia-induced accumulation of HIF-1α and its mitophagy target BNIP3. BNIP3 downregulation resulted in a significant reduction of cell viability, suggesting a cytoprotective role of mitophagy in our HCC *in vitro* model. Melatonin addition was able to abolish HIF-1α-induced mitophagy and enhance apoptotic cell death through the inhibition of HIF-1α/BNIP3 axis, thus improving sorafenib efficacy.

These results suggest that melatonin can efficiently suppress the prosurvival HIF-1α-induced mitophagy, becoming a potential coadjuvant for the chemotherapeutic treatment of HCC.

Funding:

CIBERehd is funded by Instituto de Salud Carlos III. FF and NPD are supported by the Ministry of Education of Spain (Becas FPU: FPU16/05277 and FPU13/04173, respectively), CMB by the Asociación Española Contra el Cáncer (AECC)-Junta provincial de León, and PFP by the IBIOMED-University of León.

## P4 Bibliographic review of cellular differentiation of ommatidium in *Drosophila melanogaster*

### Pablo González-García, Jose M. Bellido-Gutiérrez

#### Department of Biotechnology, Biomedicine and Public Health, University of Cádiz, Puerto Real, 11510, Spain

##### **Correspondence:** Pablo González-García (pagongar_98@hotmail.com)

*Drosophila melanogaster* has been of special importance for understanding human embryogenesis at genetic and molecular level, due to the notable similarities between their genome and ours (and, in this case, their development mechanisms), the amount of knowledge there is about this species, and many other reasons that make the *Drosophila melanogaster* an excellent model organism. In particular, cell signalling pathways involved in cell and tissue differentiation studied in said organism have shed light on biochemical mechanisms responsible for human embryonic development. With this bibliographic review, we intend to show the different signalling pathways involved in the formation of the compound eyes (and, therefore, of the ommatidia they are made of) of the dipteran during its embryogenesis. The most important pathways in this process are mainly three, all of them related with transmembrane receptors. The first of them is Spitz/DER, in which the ligand, Spitz, is secreted by adjacent cells to a precursor cell. This cell contains a transmembrane protein called DER, with intrinsic tyrosine kinase activity, which promotes differentiation. A second pathway of special interest is the Boss/Sev (Bridge of Sevenless/Sevenless), which is activated when a transmembrane proteic ligand (Boss), situated in the precursor cell’s membrane, causes conformational changes in Sevenless, a Receptor Tyrosine Kinase (RTK) on the membrane of another precursor cell, which then goes through the differentiation process. And the third pathway is the Notch/Delta. The same way as in Spitz/DER, both Notch and Delta are transmembrane proteins located in adjacent cells. In this case, the signalling pathway starts with several proteolytic cleavages. In essence, all these pathways coordinate to promote the development of the ommatidium. Finally, we will highlight some of the genes that codify the main pigments that give Drosophila melanogaster’s compound eyes their characteristic red coloration. Likewise, we will show the consequences that mutations in these loci can have, and how this causes vastly different colours in the pigmentary cells of the ommatidia.

References

1. Kuman J P, *et al.* Building an ommatidium one cell at a time. Dev Dyn. 2012; 241: 136–149.

2. Ben-Zion S, *et al*. Signaling by the Drosophila epidermal growth factor receptor pathway during development. Cell Research. 2003; 284: 140–149.

3. Matthew F, *et al*. Reiterative use of the EGF receptor triggers differentiation of all cell types in the Drosophila eye. Cell Press. 1996; 87: 651–660.

4. Baonza A, Matthew F. Notch signalling and the initiation of neural development in the Drosophila eye. Development. 2001; 128: 3889-3898.

## P5 The role of FurC in the response of *Anabaena* PCC7120 to oxidative stress

### Guillermo Nevot, Cristina Sarasa, Andrés González, María L. Peleato, María F. Fillat, Emma Sevilla

#### Department of Biochemistry and Molecular and Cell Biology and Institute for Biocomputation and Physics of Complex Systems, University of Zaragoza, Pedro Cerbuna 12, 50009-Zaragoza, Spain

##### **Correspondence:** Emma Sevilla (esevilla@unizar.es)

Fur proteins are a widespread family of transcriptional regulators present in a broad range of bacteria, some of them responsible for pathogenicity. In the cyanobacteria *Anabaena* PCC7120, three paralogs of this family have been identified as FurA, FurB and FurC. Whereas FurA and FurB have defined roles as iron and zinc regulators, the function of FurC remains unclear. Previous research in our group revealed that FurC was unable to bind any of the three *fur* promoters. Nevertheless, FurC was shown to modulate FurA and FurB autoregulation *in vitro*, intensifying DNA binding of the former and inhibiting the latter [1]. Recently, the strong induction of FurC expression under oxidative stress conditions and its action mechanism has led to purpose it as the hydrogen peroxide regulator (PerR) of *Anabaena* PCC7120 [2]. In the present study, we have analysed the effects of FurC-overexpression in the phenotype and transcriptional profile of *Anabaena* PCC7120 to provide further insights in FurC regulation. Interestingly, the high levels of FurC led to more sensitivity to oxidative stress produced either by hydrogen peroxide or methyl viologen. Additional biochemical assays showed a reduction of superoxide dismutase and catalase activities among other physiological alterations. In order to assess the behaviour of the strain under oxidative stress, the expression of key genes involved in this response was determined. Our data revealed an impairment of the expression pattern under oxidative conditions in the genes *sodA* (superoxide dismutase), *alr0998* (Mn-catalase) and *prxA* (peroxiredoxin), suggesting that FurC might directly or indirectly regulate them. To prove this, EMSA analysis were performed with FurC protein and *prxA, sodA* and *alr0998* promoters showing FurC ability to bind only to *prxA* promoter. However, FurB protein was previously described in our laboratory to bind *alr0998* and *sodA* promoters, regulating their transcription. In this context, we repeated EMSA assays incubating FurB and FurC together and the results suggested an undescribed regulatory mechanism involving these two proteins that could indicate a crosstalk between oxidative stress and zinc homeostasis. Taken together, our results reveal that the role of FurC in oxidative stress response is more complex than it was previously expected.

References

1. Hernández J A, López-Gomollón S, Bes M T, Fillat M F, Peleato M L. Three fur homologues from *Anabaena sp.* PCC7120: exploring reciprocal protein-promoter recognition. FEMS Microbiol Lett. 2004; 236(2):275–82.

2. Yingping F, Lemeille S, Talla E, Janicki A, Denis Y, Zhang C-C, *et al*. Unravelling the cross-talk between iron starvation and oxidative stress responses highlights the key role of PerR (alr0957) in peroxide signalling in the cyanobacterium *Nostoc* PCC 7120. Environ Microbiol Rep. 2014; 6(5):468–75.

Funding:

BFU2016-77671-P

## P6 UNC0642 epigenetically activates autophagy and modulates the cellular characteristics and survival of U2OS cells

### Ignacio Rodriguez-Pastrana^1^, Amanda S. Coutts^2^

#### ^1^ School of Science and technology, Nottingham Trent University, Clifton Campus, Nottingham, Nottinghamshire, NG11 8NS, United Kingdom; ^2^ John van Geest Cancer Research Centre - School of Science and technology, Nottingham Trent University, Clifton Campus, Nottingham, Nottinghamshire, NG11 8NS, United Kingdom

##### **Correspondence:** Ignacio Rodriguez-Pastrana (rodpas@ono.com)

Autophagy is a catabolic process that degrades different cytoplasmic components and damaged-organelles in order to allow the cells to reuse their basic biomolecules. Autophagy is a cellular intrinsic pathway that maintains the tissue homeostasis, but it can easily be activated by different stress stimuli like starvation or chemotherapeutic-drugs, acquiring a cell survival role. UNC0642 is an epigenetic G9a-methyltransferase inhibitor, which reduces the methylation levels of H3K9 (histone-3/lysine-9). We show that, the presence of UNC0642 produces cellular density and morphological changes with concentrations greater than 1μM-dose during 48 hours of exposure, with a possible senescence induction. Furthermore, we report that UNC0642 presents low toxicity levels being the first epigenetic G9a-methyltransferase inhibitor to be tested in vivo. We also show that, UNC0642 rises in a time and concentration-dependent manner the autophagy process. This autophagy induction is demonstrated by an increase in the transformation of LC3-I into LC3-II, a reduction of the cytoplasmic p62 levels and the accumulation of fluorescence autophagosomes. Thus, our results establish that UNC0642 is a potent epigenetic regulator, with low cytotoxicity and it can induce autophagy in osteosarcoma cells (U2OS).

References

1. Klionsky D J, *et al*. Autophagy as a regulated pathway of cellular degradation. Science. 2000; 290: 1717–1721.

2. Coutts A S, *et al*. Actin nucleation by WH2 domains at the autophagosome. Nat Commun. 2015; 6.

3. Liu F, *et al*. Discovery of an in vivo chemical probe of the lysine methyltransferases G9a and GLP. J. Med. Chem. 2013; 56: 8931–8942.

4. Yuan Y, *et al*. A Small-Molecule Probe of the Histone Methyltransferase G9a Induces Cellular Senescence in Pancreatic Adenocarcinoma. ACS Chem. Biol. 2012; 7: 1152–1157.

Funding:

NOTTINGHAM TRENT UNIVERSITY - COLLEGE OF SCIENCE AND TECHNOLOGY. Dr. AMANDA S. COUTTS

## P7 Characterization of *Klebsiella pneumoniae* bacteriophages with biotechnological potential

### Beatriz Torres^1^, Sailee Shorff^3^, Katariina Koskinen^2^, Reetta Penttinen^2^, Matti Jalasvuori^2^

#### ^1^ University of Zaragoza, Zaragoza, 50009, Spain; ^2^ Department of Biological and Environmental Science, Nanoscience Center, University of Jyväskylä, Jyväskylä, 40014, Finland; ^3^ University of Jyväskylä, Jyväskylä, 40014, Finland

##### **Correspondence:** Beatriz Torres (beath96@gmail.com)

*Klebsiella pneumoniae* has emerged as a major cause of hospital-acquired infections. The extensive use and misuse of antibiotics has led to an increased emergence of multidrug-resistant *Klebsiella pneumoniae* strains, which are a serious concern worldwide since they have a great propensity to spread and a small number of effective treatments are left [1]. Consequently, phage therapy is garnering renewed interest as an alternative method to defeat antibiotic resistant bacteria. Alongside this, phages – natural pathogens of bacteria – have several properties, such as high capacity to replicate as long as the host is present and high host specificity that turns them into a great advantage in the face of antibiotics [2].

In this study, eight bacteriophages were characterized according to their genetic material and morphology by performing endonuclease digestions and transmission electron microscopy imaging with 1% phosphotungstic acid or 2% uranyl acetate as staining dyes. Then, they were classified in agreement with their morphological characterization.

Seven phages were classified into *Siphoviridae* family (EKP3P1, EKP3P2, EKP3P4, EKP3P5, EKP8P2, EKP8P3, EKP8P4) showing hexagonal heads with long non-contractile, sometimes flexible, tails and closely related restriction patterns among them. EKP8P1 phage was classified into *Podoviridae* family showing an icosahedral head with a short non-contractile tail and a different restriction pattern. All of them belong to *Caudovirales* order. In addition, a prophage was found in EKP8P1 sample, and classified into *Siphoviridae* family according to its morphology.

The genome of EKP3P5 phage, a double stranded DNA of 47,622 bp long, was sequenced and, then, manually annotated. EKP3P5 phage turned out to be a temperate phage encoding integrase, holin and endolysin proteins, among others. As a temperate phage, EKP3P5 could not be used in phage therapy due to the risk of transferring virulence and resistance genes to the host bacteria, turning it into a more pathogenic strain and contributing in antibiotic resistance dissemination.

For all the above reasons, this study provides detailed knowledge of the physical structure along with genomic qualities of eight multidrug-resistant *Klebsiella pneumoniae* infective bacteriophages. This is important for determining the potential of phages as therapeutic agents and the first step to improve phage therapy.

References

1. *World Health Organization*. Antimicrobial resistance: global report on surveillance. 2014

2. Matsuzaki S, *et al*. Bacteriophage therapy: a revitalized therapy against bacterial infectious diseases. J. Infect. Chemother. 2005; 11: 211–219.

## P8 Multi-stability of blood circulation in ladder-shaped networks

### Beatriz De-Vicente-Martínez, Thomas Podgorski, Gwennou Coupier

#### Laboratoire Interdisciplinaire de Physique (LiPhy) UMR5588, CNRS-Université Grenoble Alpes, Saint Martin d'Hères, Grenoble, 38402, France

##### **Correspondence:** Beatriz De-Vicente-Martínez (beatrizdevic@hotmail.com)

All human cells need oxygen supply as well as carbon dioxide removal and red blood cells (RBCs) are in charge of those functions. To make that possible, blood flows through a complex network of the circulatory system, from large arteries to very tiny capillaries where RBCs accomplish their vital functions. It is worthy to note that RBCs do not behave as passive tracers, as their size is comparable to that of blood capillaries. RBCs flexibility and dynamics have a decisive role in the haematocrit partition at the level of bifurcations [1], which is the main mechanism that dictates blood heterogeneity in the microvascular networks, and it affecting many physiological functions, although it remains poorly understood. Therefore, in order to have a better insight of some causes and consequences related to circulatory pathologies, it would be interesting to study the parameters that influence the partition of haematocrit at the microvascular bifurcations, to improve and lead to new applications in biomedical technology, for example in blood substitute development and transfusion techniques [1]. Previous computer simulations, consisting in numerical resolution of the system of equations governing the flow in ladder-shaped networks using specific models for blood rheology [2] and phase separation, predict interesting and non-intuitively multi-stability states for the velocity profile and haematocrit partition in ladder-shaped microchannels. In this study, we mimicked human capillaries utilizing microfluidic chips and studied the soundness or not of those simulations. For that, we carried out blood flow experiments through 2-rung ladder-shaped microchannels. The results prove the existence of different states and at least two branches of stable solutions in the 2-rung ladder-shaped network. Furthermore, both show the tendency to be maintained in those branches, but also an oscillatory behaviour in time. Related with the precedent *in silico* solutions, we prove the main characteristic of multiple-stability states, but the shape of the solutions discerns.

This work has been the first experimental characterisation of multi-stability for blood flow in channel networks and a preliminary approach to RBCs suspensions behaviour in a basic model of capillary beds, which opens the door to new studies in the area.

References

1. Shen Z, Coupier G, Kaoui B, Polack B, Harting J, Misbah C, Podgorski T. Inversion of hematocrit partition at microfluidic bifurcations. Microvasc Res. 2016; 105: 40-46.

2. Pries A R, Neuhaus D, Gaehtgens P. Blood viscosity in tube flow: dependence on diameter and hematocrit. Am J Physiol. 1992; 363: 6135-6192

## P9 Synthesis and evaluation of the biological activity of lipopeptides derived from BP100 containing a D-amino acid

### Lluis Moll^1^, Àngel Oliveras^1^, Lidia Feliu^1^, Marta Planas^1^, Esther Badosa^2^, Emilio Montesinos^2^

#### ^1^ LIPPSO, Department of Chemistry, University of Girona, Girona, 17003, Spain; ^2^ Laboratory of Plant Pathology, Institute of Food and Agricultural Technology-CIDSAV-XaRTA, University of Girona, Girona, 17003, Spain

##### **Correspondence:** Lluis Moll (lluismds@hotmail.com); Lidia Feliu (lidia.feliu@udg.edu)

Nowadays, world population has reached 7,6 billion and it is expected to rise up to 11,2 billion in 2100. This increase requires to step up the agricultural yield in order to supply food for the whole population. A way to achieve this purpose would be reducing the losses caused by microorganisms [1]. Recent restrictions imposed by the European Union on the use of several compounds to combat these pathogens, has prompted the search for safer alternatives. Antimicrobial peptides are considered as optimal candidates.

Some years ago, our group, in collaboration with the Laboratory of Plant Pathology, described the lead peptide KKLFKKILKYL-NH_2_ (BP100) with antimicrobial activity against economically important plant pathogens [2]. More recently, with the aim of obtaining peptides with better biological properties, several lipopeptides have been synthetized. This type of peptides contains a fatty acid in their structure to favour their insertion into the pathogen membrane [3]. The lipopeptides derived from BP100 displayed high antimicrobial activity, but they were hemolytic and poorly stable.

In this work, in order to enhance the properties of the above BP100 derivatives, new lipopeptides have been described. In particular, the amino acid at position 4 has been replaced by its D enantiomer. This approach has been designed to reduce the hemolysis and to increase the stability, while maintaining the antimicrobial activity [4]. These new lipopeptides have been prepared on solid phase. They have been analysed by HPLC and characterized by ESI-MS. They have been tested against the phytopathogenic bacteria *Erwinia amylovora, Pseudomonas syringae pv. syringae*, *Pseudomonas syringae pv. actinidiae, Xanthomonas arboricola pv. pruni, Xanthomonas fragariae, Xantomonas axonopodis pv. vesicatoria,* and the fungi *Penicillium expansum* and *Fusarium oxysporum*. Furthermore, the toxicity and the stability have been analysed. The best lipopeptides will be selected to be assayed *in* vivo and, if they are successful, they will be produced in plants to decrease the production cost.

References

1. Savary S, Ficke A, Aubertot J N, Hollier C. Crop Losses Due to Diseases and Their Implications for Global Food Production Losses and Food Security. Food Secur. 2012; 4:519-537

2. Badosa E, Ferre R, Planas M, Feliu L, Besalú E, Cabrefiga J, Bardají E, Montesinos E. A Library of Linear Undecapeptides with Bactericidal Activity against Phytopathogenic Bacteria. Peptides. 2007; 28:2276-2285.

3. Malina A, Shai Y. Conjugation of Fatty Acids with Different Lenghts Modulates the Antibacterial and Antifungal Activity of Cationic Biologically Inactive Peptide. Biochem J. 2005; 390:695-702.

4. Guell I, Cabrefiga J, Badosa E, Ferre R, Talleda M, Bardají E, Planas M, Feliu L, Montesinos E. Improvement of the Efficacy of Linear Undecapeptides against Plant-Pathogenic Bacteria by Incorporation of D-Amino Acids. Appl Enviorn Mirobiol. 2011; 77:2667-2675.

Funding:

MINECO, Grant AGL2015-69876-C2-2-R; University of Girona, MPCUdG2016/038

## P10 Seminal plasma from boar ejaculates with high resistance to cold shock could improve post-thawing sperm quality

### Andrea Núñez-González^1^, Itxaso Crespo-Félez^1,2^, Estela Fernández-Alegre^1,2^, Touba Nadri^3^, Felipe Martínez-Pastor^1,2,4^

#### ^1^ INDEGSAL, León, 24071, Spain; ^2^ Grupo IMAPOR, León, 24071, Spain; ^3^ Department of Animal Science, College of Agriculture and Natural Resources, University of Tehran, Karaj, Iran; ^4^ Department of Molecular Biology, Universidad de León, León, 24071, Spain

##### **Correspondence:** Andrea Núñez-González (annugo95@gmail.com)

Swine industry depends on the generation of seminal doses of selected boars and their application by artificial insemination, which is mainly carried out by using refrigerated semen. As boar spermatozoa are particularly sensitive to cryopreservation, frozen doses still produce variable results [1]. When sperm is cold-shocked by rapid cooling, the resulting injuries do not present the same intensity in every animal. This variability may be due not only to intrinsic differences in the sperm membranes but also to the action of external factors such as seminal plasma (SP). It is well-known that quantitative differences in specific SP components exist between sires. Hence, the protein composition of SP could explain differences between boars regarding the ability of their sperm to sustain cryopreservation. This study aimed to evaluate the effects of boar SP on membrane integrity, acrosomal status, mitochondrial activity and capacitation of spermatozoa after thawing, presuming that its effectiveness depends on the male from which it is obtained. We proposed that SP derived from males whose semen is resistant to cold shock could be the most suitable for its application as a protector. Thus, this study was conducted to compare the SP of males producing semen with different resistance to cold shock, in terms of its effectiveness in maintaining the quality of thawed sperm. Our work evidenced that the addition of 20 % (v/v) SP arising from cold shock-resistant boars increases post-thaw motility relative to non-supplemented sperm (p<0.001). Remarkably, this effect was not evident when using SP from boars whose semen was classified as susceptible to cold shock. Besides, supplementation with SP, regardless its origin, had some detrimental effects on spermatozoa (increased apoptosis rate, capacitation status and acrosomal damage), but it improved (p<0.05) sperm viability after thawing relative to control sperm. Our results show that the addition of SP to frozen-thawed seminal doses induces changes in sperm physiology, presumably through the interaction of plasma proteins with the sperm membrane. Those changes are more desirable when SP comes from males classified as highly-resistant, evidencing that cold shock could be a useful tool when selecting boars from which SP should be obtained.

Reference

1. Fernández-Gago R, Álvarez-Rodríguez M, Alonso M E, Ramiro-González J, Alegre B, Domínguez J C, Martínez-Pastor F. Thawing boar semen in the presence of seminal plasma improves motility, modifies subpopulation patterns and reduces chromatin alterations. Reprod Fertil Dev. 2017; 29(8):1576-1584.

Funding:

This work has been supported by project CEI15-24 (Ministry of Education, Culture and Sport, Spain), through Campus of International Excellence of the University of Oviedo. The authors thank Topigs-Norsvin Ibérica (León, Spain) for housing the animals and providing the semen samples.

